# Interrupted time series design to evaluate the effect of the ICD-9-CM to ICD-10-CM coding transition on injury hospitalization trends

**DOI:** 10.1186/s40621-018-0165-8

**Published:** 2018-10-01

**Authors:** Svetla Slavova, Julia F. Costich, Huong Luu, Judith Fields, Barbara A. Gabella, Sergey Tarima, Terry L. Bunn

**Affiliations:** 1 Department of Biostatistics, College of Public Health, University of Kentucky, Lexington, KY USA; 2 Kentucky Injury Prevention and Research Center, University of Kentucky, Lexington, KY USA; 3 Department of Health Management and Policy, College of Public Health, University of Kentucky, Lexington, KY USA; 4 Colorado Department of Public Health and Environment, Denver, CO USA; 5 Institute for Health and Equity, Medical College of Wisconsin, Milwaukee, WI USA; 6 Department of Preventive Medicine and Environmental Health, College of Public Health, University of Kentucky, Lexington, USA

## Abstract

**Background:**

Implementation of the International Classification of Diseases, Tenth Revision, Clinical Modification (ICD-10-CM) in the U.S. on October 1, 2015 was a significant policy change with the potential to affect established injury morbidity trends. This study used data from a single state to demonstrate 1) the use of a statistical method to estimate the effect of this coding transition on injury hospitalization trends, and 2) interpretation of significant changes in injury trends in the context of the structural and conceptual differences between ICD-9-CM and ICD-10-CM, the new ICD-10-CM-specific coding guidelines, and proposed ICD-10-CM-based framework for reporting of injuries by intent and mechanism. Segmented regression analysis was used for statistical modeling of interrupted time series monthly data to evaluate the effect of the transition to ICD-10-CM on Kentucky hospitalizations’ external-cause-of-injury completeness (percentage of records with principal injury diagnoses supplemented with external-cause-of-injury codes), as well as injury hospitalization trends by intent or mechanism, January 2012–December 2017.

**Results:**

The segmented regression analysis showed an immediate significant drop in external-cause-of-injury completeness during the transition month, but returned to its pre-transition levels in November 2015. There was a significant immediate change in the percentage of injury hospitalizations coded for unintentional (3.34%) and undetermined intent (− 3.39%). There were immediate significant changes in the level of injury hospitalization rates due to poisoning, suffocation, struck by/against, other transportation, unspecified mechanism, and other specified not elsewhere classifiable mechanism. Significant change in slope after the transition (without immediate level change) was identified for assault, firearm, cut/pierce, and motor vehicle traffic injury rates. The observed trend changes reflected structural and conceptual features of ICD-10-CM coding (e.g., poisoning and suffocations are now captured via diagnosis codes only), new coding guidelines (e.g., requiring coding of injury intent as “accidental” if it is unknown or unspecified), and CDC proposed external-cause-of-injury code groupings by injury intent and mechanism. Researchers can replicate this methodology assessing trends in injuries or other ICD-10-CM-coded conditions using administrative billing data sets.

**Conclusions:**

The CDC ‘s *Proposed Framework for Presenting Injury Data Using ICD-10-CM External Cause of Injury Codes* provided a logical transition from the ICD-9-CM-based matrix on injury hospitalization trends by intent and mechanism. Our findings are intended to raise awareness that changes in the ICD-10-CM coding system must be understood to assure accurate interpretation of injury trends.

**Electronic supplementary material:**

The online version of this article (10.1186/s40621-018-0165-8) contains supplementary material, which is available to authorized users.

## Background

On October 1, 2015, diagnostic coding in U.S. inpatient and outpatient administrative billing data transitioned from the International Classification of Diseases, Ninth Revision, Clinical Modification (ICD-9-CM) to ICD-10-CM, long after other countries with developed industrial economy and high per capita income (e.g., Germany, Australia, Canada) had implemented ICD-10-based morbidity systems. The U.S. transition was motivated by limitations in the ICD-9-CM coding system to accurately describe current clinical practices and patients’ medical conditions, by barriers to direct comparison between ICD-9-CM-coded U.S. morbidity data and ICD-10-coded U.S. mortality data, and by challenges to comparing U.S. and international morbidity data (U.S. Department of Health and Human Services 2009). The number of codes increased from about 17,000 in ICD-9-CM to more than 155,000 in ICD-10-CM (Topaz et al. 2013).

A major argument in support of the ICD-10-CM system was its increased level of coding detail for traumatic injuries. The injury codes in ICD-10-CM constitute about 60% of all codes compared with about 15% in ICD-9-CM (Topaz et al. 2013). Studies of the transition from ICD-9-CM to ICD-10-CM reported a significant increase in medical coding time and a decrease in medical coder productivity, especially for inpatient coding (Weems et al. 2015).

External cause of injury (ECOI) codes capture information on injury cause (e.g., fall, poisoning, or motor vehicle crash) and injury intent (unintentional/accidental, self-harm, assault, or undetermined). Information derived from ECOI coding is the basis for injury surveillance and epidemiology, and informs targeted injury prevention planning and evaluation. However, ECOI codes are not required for health care provider billing and reimbursement, raising a concern that increased ICD-10-CM time demands and decreased medical coder productivity could discourage coders from entering ECOI codes for injury-related encounters of care.

The transition from ICD-9-CM to ICD-10-CM coding also has the potential to affect epidemiological analysis of injury morbidity trends (Fenton and Benigni 2014; Injury Surveillance Workgroup 9 2016). When evaluating the effect of the transition to ICD-10-CM on injury hospitalization trends, injury epidemiologists need to know where to expect significant and sustained changes in trends based on 1) structural and conceptual differences between ICD-9-CM and ICD-10-CM, 2) new ICD-10-CM-specific coding guidelines, and 3) structural differences between the ECOI matrices for reporting injuries coded in ICD-9-CM and ICD-10-CM (CDC 2011; Annest et al. 2014).

The CDC’s *Proposed Framework for Presenting Injury Data using ICD-10-CM External Cause of Injury Codes* provides definitions for injury classification by intent and mechanism of injury (Annest et al. 2014). The majority of the proposed categories are conceptually equivalent to the categories in the ICD-9-CM ECOI matrix (CDC 2011). The authors expected that if the definitions for a specific intent or injury mechanism in the two matrices are conceptually equivalent (or very close), there should be no abrupt changes in trends by injury intent or mechanism immediately after the implementation of ICD-10-CM. Significant immediate changes that are then sustained after the initial transition period may indicate conceptual or coding differences in the ICD-10-CM system affecting the magnitude of captured injury cases, or that the ICD-10-CM intent or mechanism category captured cases that belonged to a different intent or mechanism category in the ICD-9-CM ECOI matrix.

This study evaluated the effect of the transition from ICD-9-CM to ICD-10-CM on the completeness of ECOI codes and the significance of changes in injury hospitalization trends by intent of injury and by mechanism of injury, using Kentucky statewide inpatient hospital discharge administrative billing records.

## Methods

### Data

The analysis used Kentucky statewide inpatient hospital discharge administrative billing data for Kentucky residents treated in Kentucky acute care hospitals between January 1, 2012 and December 31, 2017. Data were provided by the Kentucky Office of Health Policy, Cabinet for Health and Family Services. State data policy requires the removal of personal identifiers from state administrative billing data sets (see Kentucky Revised Statutes 216.2927). Thus, transfers from one hospital to another or readmissions for active treatment of the same injury could not be identified. Data presented here reflect instances of inpatient hospitalizations, rather than distinct injured patients or injuries. A hospital injury data set was extracted based on a principal diagnosis of injury. The selection of injury diagnoses was based on the CDC *State Injury Indicators Report: Instructions for Preparing 2015 Data,* for ICD-9-CM-coded data (Thomas and Johnson 2017); and CDC’s *Proposed Framework for Presenting Injury Data using ICD-10-CM External Cause of Injury Codes,* for ICD-10-CM-coded data (Annest et al. 2014).

### Measures

This study defined three injury hospitalization measures used as model outcome variables.

1. The ECOI completeness was defined as the percentage of records with a principal diagnosis of injury that were supplemented with ECOI codes specifying the mechanism/cause and intent of injury. The ECOI completeness measure was calculated for each month during the study period, forming a time series of 72 observations.
2. Injury intent was identified for each injury hospitalization record with an ECOI code (CDC 2011; Annest et al. 2014; CDC/NCHS 2016). Monthly measures “percentage of injury hospitalizations by intent” (unintentional, intentional self-harm, assault, and undermined intent) were calculated.
3. Injury mechanism (cause of injury) was identified for each injury hospitalization record with a valid ECOI (CDC 2011; Annest et al. 2014). Monthly crude rate (per 100,000 population) was calculated for each injury mechanism.

### Analysis

The transition to ICD-10-CM on October 1, 2015, was a significant policy change (intervention) that could have affected (interrupted) the previously established trend of ECOI completeness as well as the injury hospitalization measures of cause and intent. Segmented regression analysis is one method for statistical modeling of an interrupted time series data and it was used in this study to evaluate the impact of the transition to ICD-10-CM coding on the monitored trend of a specific injury hospitalization measure (Wagner et al. 2002; Bernal et al. 2017). We viewed the ICD-10-CM transition as an intervention that split the time series of an injury hospitalization measure. The trend within each segment can be approximated by a linear regression line, defined by two parameters: level and slope. The level is the value (intercept) of the time series at the beginning of a time interval (segment). The slope is the trend, i.e. the average rate of monthly change during the time segment. Each segment has its own intercept and slope. Segmented regression analysis is used to evaluate simultaneously both the significance of the immediate change in the injury hospitalization measure after the coding transition (immediate intervention effect), and the increase or decrease in the slope of the regression line after the transition in comparison to the trend/slope of the regression line before the transition (change in trend). We used the following model:

$$ {Y}_{\mathrm{t}}={\beta}_0+{\beta_1}^{\ast }{time}_t+{\beta_2}^{\ast } ICD10{CM}_t+{\beta_3}^{\ast } time- after- ICD10{CM}_t+{\varepsilon}_t, $$

where *Y*_t_ is the injury hospitalization measure in month *t*, with t taking values from 1 (for January 2012) to 72 (for December 2017).The first segment, before the transition, is represented by the intercept *β*_*0*_ (baseline level of the injury hospitalization measure at time *t* = 0, December 2011) and the slope *β*_*1*_ (average monthly change in the injury hospitalization measure before the transition to ICD-10-CM). The variable *ICD10CM* is equal to 0 before October 1, 2015 and equal to 1 for observations on or after October 2015. The variable *time-after-ICD10CM* measures the time units after the intervention (0 for January 2012 to September 2015), and takes values from 1 to 27 for October 2015 to December 2017). The coefficient *β*_*2*_ represents the immediate effect of the transition, i.e., the change in level (drop or jump) of the injury hospitalization measure immediately after the transition. The coefficient *β*_*3*_ represents the change in the slope (increase or decrease) of the monthly injury hospitalization measure after the transition to ICD-10-CM, compared to the slope *β*_*1*_ for the segment before the transition. Thus, *β*_*1*_ + *β*_*3*_ represents the post-intervention slope of the segment after the transition, i.e., the average percentage increase in the injury hospitalization measure from 1 month to the next after the transition to the ICD-10-CM coding. The error term *ɛ*_*t*_ represents the random variability not explained by the variables in model (e.g., unaccounted seasonal variations, data quality anomalies).

One assumption of the ordinary least squares regression analysis is that error terms are uncorrelated. When this assumption is violated, statistical tests for the significance of the parameters are not correct. The autoregressive error model can correct for autocorrelation. The general form of the autoregressive error model of order *k* would be
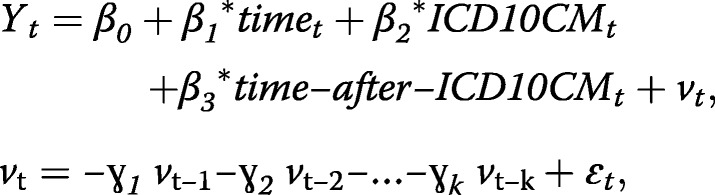


where *ɛ*_*t*_ are independently normally distributed with mean zero and variance *σ*^2^*.*

Proc AUTOREG with a BACKSTEP option was used to select the correct order of the autoregressive error model. Since seasonality produces autocorrelation at the seasonal lag, it is recommended that the initial full model has an order equal to or larger than the order of any potential seasonality (SAS/ETS(R) 9.3 User’s Guide 2018; Penfold and Zhang 2013). In our monthly models, we specified NLAG = 12 in the stepwise autoregression. The AUTOREG procedure automatically tests for correlations and estimates the autoregressive parameters. Backward elimination sequentially removes autoregressive parameters not significant at the 0.05 level. Sensitivity analyses were performed using a less restrictive threshold at the 0.1 level (SLSTAY = 0.1). The stepwise autoregressive process in the AUTOREG procedure is performed using the Yule-Walker method. The maximum likelihood estimates are produced after the order of the model is determined from the significance test of the preliminary Yule-Walker estimates (SAS/ETS(R) 9.3 User’s Guide 2018). The Fit Diagnostic panel (proc AUTOREG PLOTS = all) for each model outcome (injury hospitalization measure) was reviewed to evaluate model assumptions and fit, inspecting the residuals plot, the white noise probability plot, the autocorrelation function (ACF) plot, and the partial autocorrelation function (PACF) plot (Chvosta 2009).

Initial inspection of the quality and completeness of the Kentucky injury hospitalization billing records exposed a significant drop (about 10%) in the percentage of the ECOI completeness in April, May, and June of 2013. During this period, ECOI completeness for the hospital with the largest volume of injury cases in Kentucky fell from above 95% to about 50%. The decline in this hospital’s ECOI completeness alone accounted for a drop of over 5% in the total state ECOI completeness. Three other relatively large volume hospitals had between 10% to 20% declines in ECOI completeness during the same period, adding a cumulative 3% to the statewide drop. So, the 10% drop in the statewide ECOI completeness can be explained as a data quality/reporting issue, and the ECOI measurements during April–June, 2013 are outliers. ECOI reporting is neither required for billing reimbursement nor mandated in Kentucky. Therefore, these are plausible observations reflecting the reality of billing data collection, particularly during changes in coder staffing and other transitions. In order to isolate the effect of this quarter’s data quality on the parameter estimates in our models, we included an indicator variable that had a value of 1 for the months April – June, 2013, and values of 0 for all other months. If the parameter estimate for the data quality issue was not significantly different from zero, the indicator variable was excluded from the final model.

For each modeled injury hospitalization measure of interest, we reported the maximum likelihood regression coefficient estimates from the final model. When the backward elimination of autoregressive terms resulted in a final model that contained autoregressive terms, we reported the parameters of interest from the SAS AUTOREG procedure’s final model parameter estimates with “autoregressive parameters assumed given” (see Additional file 1 for an example). We reported only the parameters of interest for *time* (an important control for overall secular trend in the modeled outcome), *ICD10CM* (level change in modeled outcome after the transition to ICD-10-CM coding), and *time-after-ICD10CM* (slope change in trend line after the transition to ICD-10-CM compared to slope of the trend line before the transition).

**Fig. 1:**
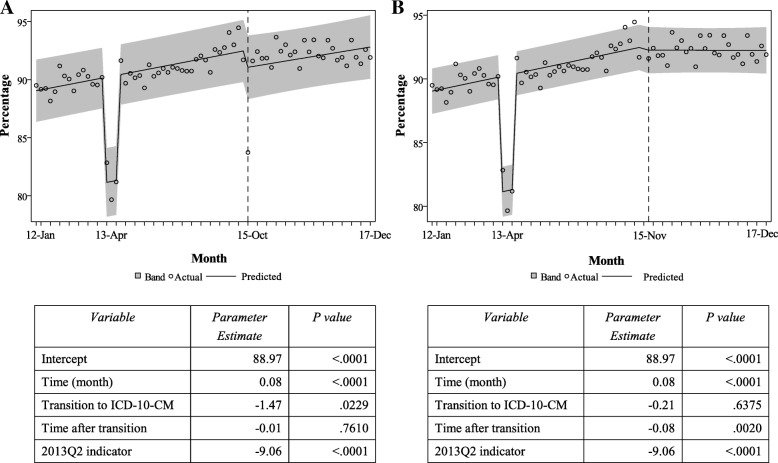
**a** Percentage of External-Cause-of-Injury (ECOI) completeness in injury hospitalization data, Kentucky resident inpatient hospitalizations, January 2012 – December 2017 (72 monthly observations). **b** Percentage of ECOI completeness in injury hospitalization data, Kentucky resident inpatient hospitalizations, January 2012– December 2017, (October 2015 observation removed; 71 monthly observations)

**Fig. 2:**
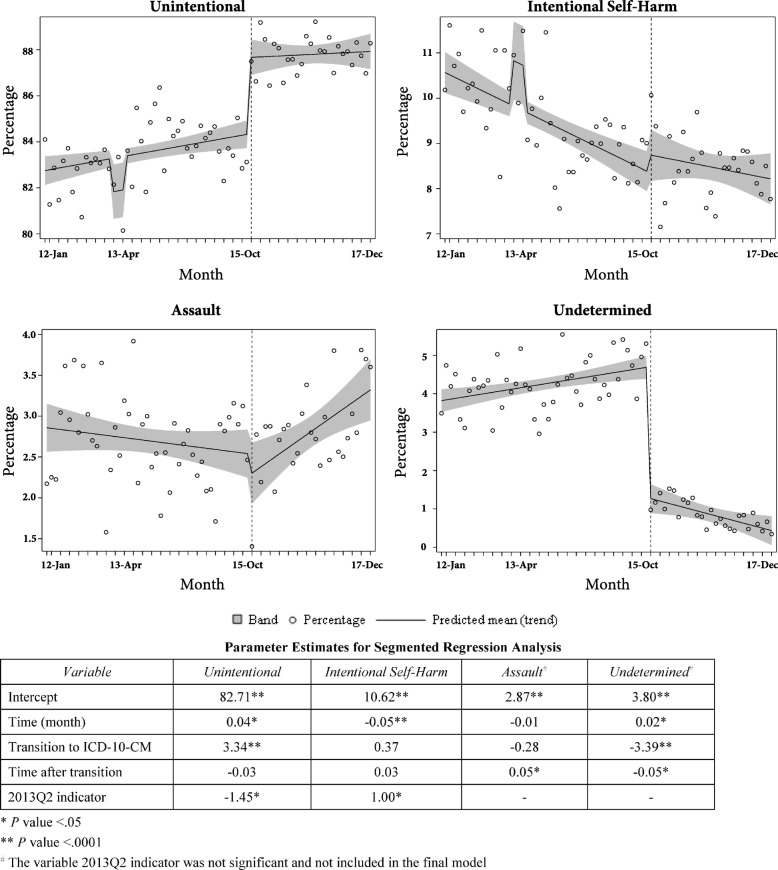
Kentucky Resident Injury Hospitalizations by Intent of Injury, January 2012 – December 2017

**Fig. 3:**
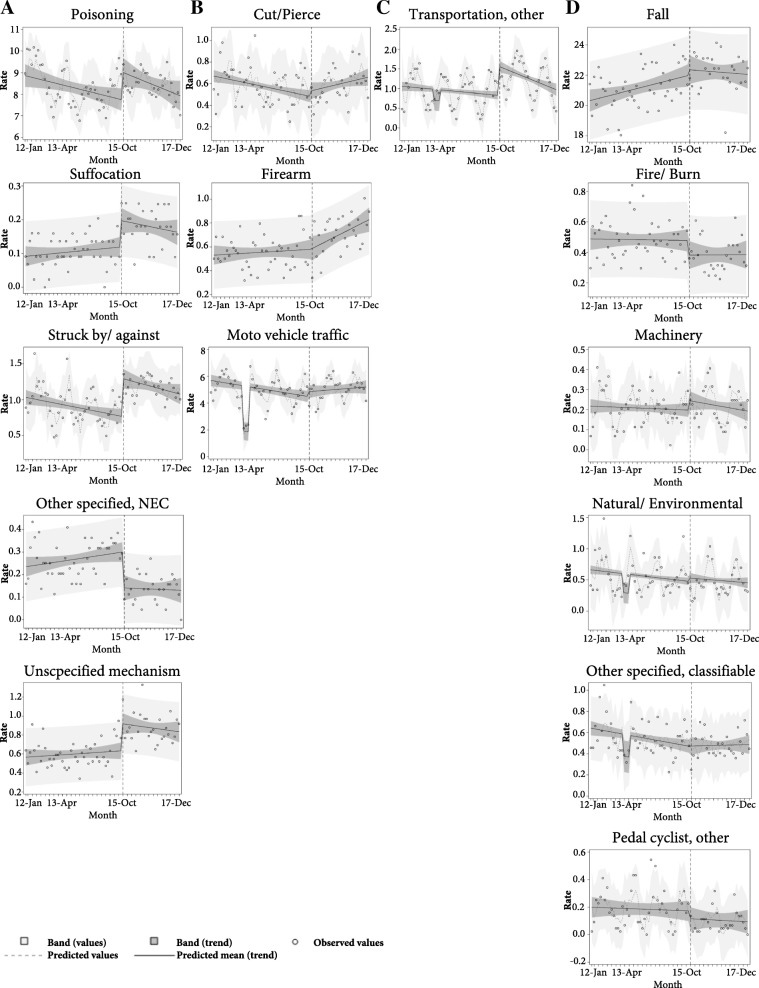
Trends in injury hospitalization rates per 100,000 population, by pattern of observed change after the transition to ICD-10-CM coding, and by injury mechanism, Kentucky resident inpatient hospitalizations, January 2012 – December 2017: **a** Significant level change without significant slope change. **b** Significant slope change without significant level change. **c** Significant level and slope changes. **d** No significant changes in level or slope

The estimated trends for each injury hospitalization measure were described visually (Figs. 1, 2 and 3) by the observed values of the modeled measure, estimated trend, and 95% confidence limits for estimated trend (UCLM = and LCLM = options in the OUTPUT statement).

Formal trend analysis was not performed for “drowning” and “pedestrian, other” mechanisms due to very small counts. A different rationale led to the omission of formal trend analysis for injuries due to the “overexertion” mechanism. Overexertion codes were omitted in the ICD-10-CM version 2015 that came into effect on October 1, 2015, so there was no overexertion ICD-10-CM code between October 1, 2015 and September 30, 2016. The code X50 (overexertion and strenuous or repetitive movements) became available for use on October 1, 2016. The Proposed ICD-10-CM External Cause of Injury Matrix includes injuries coded as W18.4, “slipping, tripping and stumbling without falling”, in the mechanism “overexertion”. Inclusion of these injuries gave the impression of a significant temporary drop in overexertion injuries, whereas in reality, overexertion injuries were not captured with a dedicated code between October 1, 2015 and September 30, 2016.

While immediate changes in the injury trends after the transition to ICD-10-CM were expected and could be explained (in most cases) with the new ICD-10-CM coding guidelines, consistent increases/decreases in long-term injury trends after the transition could indicate true change in the injury incidence. Examining such trends, as a sensitivity analysis, models with different join points for the segmented regression were compared to identify statistically if the initial point for sustained changes in injury hospitalization trends preceded October 2015. Models with different join points were compared based on the maximum likelihood estimates for the Akaike’s Information Criterion (AIC) and the corrected Akaike’s information criterion (AICC) (the model with the lowest index was preferred).

The study was approved by the University of Kentucky Institutional Review Board as part of the Kentucky Injury Surveillance Quality Improvement program.

## Results

### Changes in percentage of ECOI completeness

Results of the segmented regression analysis showed that 1) the estimated coefficient for the variable *ICD10CM* was − 1.47 (*p* = 0.02), meaning there was an immediate significant drop of about 1.5% in ECOI completeness after the transition to ICD-10-CM coding; and 2) the estimated difference between the slopes before and after the transition was − 0.01% (*p* = 0.76) meaning the average monthly change in ECOI completeness after the transition was not significantly different from the monthly change before the transition (Fig. 1a). The estimated effect of the data quality issues in 2013Q2 was a drop in the ECOI completeness of about 9%, but the effect of this period on the overall trend was isolated. There was no autocorrelation or partial autocorrelation as assessed by the ACF and PACF diagnostics.

As the reader will notice in Fig. 1a, the observed completeness of ECOI coding dropped notably in October 2015 (from 91.7% in September 2015 to 83.7% in October 2015) but bounced back in November 2015 (91.6%). The October 2015 observation was very influential (Studentized residual of − 6). We repeated the segmented regression analysis removing this time point. The results from the refitted model indicated that there was no significant level change (*p* = 0.64) and there was a significant change in the slopes of the lines before and after the transition (*p* = 0.002) (Fig. 1b). Before the transition, ECOI completeness increased on average about 0.08% per month. The estimated slope after the transition (ignoring the influence of the October 2015 drop in ECOI completeness) is practically zero. In other words, if we ignore the temporary drop in ECOI completeness during the transitional month of October 2015, we can say that the trend in ECOI completeness remained steady (leveled) at the pre-transition level until the end of the study period. The model fit diagnostics (residual plot, white noise probability bar plot, ACF and PACF plots) showed adequate model fit, with no autocorrelation issues after the backward elimination of all autoregressive terms.

### Changes in injury hospitalization trends by intent of injury

The percentage of reported **unintentional** injury hospitalizations changed from 83.1% in September 2015 to 87.5% in October 2015 (data not shown). Segmented regression analysis showed a significant immediate increase (3.34%) after the transition to ICD-10-CM-coding in the percentage of injury hospitalizations coded as unintentional (Fig. 2). The percentage of unintentional injuries was increasing significantly at an average rate of about 0.04% per month before the transition to ICD-10-CM; the slope after the transition did not change significantly and was estimated as a 0.01% increase per month.

The estimated immediate effect of the coding change on the percentage of injury hospitalizations coded as **undetermined intent** was − 3.39%, indicating a significant immediate drop that mirrored the increase in injuries coded as unintentional (Fig. 2). There was also a significant difference between the regression line slopes before and after the transition (an estimated average increase of 0.02% per month before transition; an estimated slope of − 0.03% after transition).

There was no statistically significant immediate change after the transition to ICD-10-CM in percentages of injury records coded as **intentional self-harm**. Similarly, no significant differences between the slopes of the trend lines before and after the transition were observed for percentages of intentional self-inflicted injuries (Fig. 2).

There was no immediate statistically significant change after the transition to ICD-10-CM in percentages of injury records coded as **assault** (Fig. 2). However, the slope of the line after October 2015 indicated a significant change in the trend before and after the transition, with a consistent increase in the percentage of assault injuries after the transition. Such steady change in the percentage of injuries due to assault was not expected based on the proposed matrix for reporting of ICD-10-CM-coded injuries or the ICD-10-CM coding guidelines. Performing sensitivity analysis we refitted the segmented regression model to the actual counts of assault injuries and identified the same trend (Additional file 2). However, visual inspection of the observed counts of assault injuries suggested that the increase started before October 2015. Using the maximum likelihood estimates for AIC and AICC, we compared segmented regression models with different join point for the two segmented regression lines. The model with the smallest AIC and AICC was the model with a join point in March 2015 (Additional file 2). The model suggested that the number of the assault injuries was declining significantly before March 2015 but started increasing after that. It is plausible that the consistent increase in the assault injury percentage after October 2015 is a reflection of a change in the assault injury incidence that started earlier that year.

### Changes in injury hospitalization trends by mechanism of injury

There were four distinct patterns of change observed in injury hospitalization trends by mechanism after the transition to ICD-10-CM (Fig. 3, Table 1):

**Table 1:** Parameter estimates for segmented regression analysis of monthly injury hospitalization rates, January 2012–December 2017, by mechanism of injury

Mechanism of Injury	Time	ICD10CM	Time-after-ICD10CM
Cut/Pierce^a^	− 0.004*	0.05	0.01*
Fall	0.04*	0.40	−0.05
Fire/Burn	−0.0002	− 0.09	0.0003
Firearm	0.001	−0.001	0.008*
Machinery^a^	− 0.0004	0.05	−0.002
Motor Vehicle Traffic^a^	− 0.03*	0.34	0.04*
Natural/Environmental^a^	− 0.004*	0.05	0.001
Pedal cyclist, other^a^	− 0.001	−0.06	− 0.0002
Poisoning^a^	− 0.03*	1.29*	− 0.02
Struck by, against^a^	− 0.006*	0.53**	− 0.002
Suffocation	0.001	0.08*	− 0.002
Other specified and classifiable^a^	− 0.004*	0.001	0.004
Other specified, not elsewhere classifiable	0.001	−0.16**	− 0.002
Transportation, other^a^	− 0.005*	0.75**	− 0.02*
Unspecified mechanism	0.002	0.29*	− 0.005

Notes: Each model had a significant intercept

*p < 0.05

**p < 0.001

^a^The final model included autoregressive parameter(s) significant at the 0.05 level

#### Significant level change without significant slope change

In October 2015, there was an immediate significant increase in the level of injury hospitalization rates due to: 1) **poisonings** (1.29/100,000), 2) **suffocation** (0.08/100,000), 3) **struck by/against** (0.53/100,000), and 4) **unspecified mechanism** (0.29/100,000). There was an immediate significant drop in injury hospitalization rate classified as **other specified, not elsewhere classifiable** (NEC) (− 0.16/100,000). For each of these injury mechanisms, the slopes of the segmented regression lines before and after the transition were not significantly different (Fig. 3a).

A sample data set and SAS code for modeling the outcome “rate of injury hospitalizations due to poisoning” is provided in Additional file 1. The reader can compare the model fit statistics for the ordinary least square regression with the maximum likelihood estimates from the regression model with autoregressive parameters retained at level of significance 0.05.

#### Significant slope change without significant level change

Significant slope changes without significant level changes following the intervention were found for hospitalization rates due to the mechanisms cut/pierce, firearm, and motor vehicle traffic (Fig. 3b). The rate of injury hospitalizations due to **cut/pierce** injuries was declining on average by 0.004/100,000 per month before ICD-10-CM implementation; after the intervention, the average monthly rate changed significantly and was estimated as an increase of 0.006/100,000. There was no significant change in monthly hospitalization rates for **firearm** injuries before the intervention; after the coding change, the estimated change in slope was significant, with an estimated average monthly increase of 0.009/100,000. Segmented regression analysis showed that there was a significant monthly decline of 0.03/100,000 in injury hospitalization rates for **motor vehicle traffic** before the transition to ICD-10-CM coding. The estimated slope of the regression line after the transition was significantly different from the slope before the transition, with an estimated monthly increase after transition of 0.01/100,000.

#### Significant level and slope changes

There was a significant level increase in the rate due to **transportation, other** (0.75/100,000). The pre-transition trend was declining significantly; after the immediate jump in October 2015, there was a significantly steeper decline in the post-transition period (Fig. 3c).

#### No significant changes in level or slope

There was no significant immediate effect or a change in slope of the regression lines after the transition for injury hospitalization rates classified as falls, fire or burn, machinery, natural or environmental, other specified and classifiable, and pedal cyclist/other (Table 1 and Fig. 3d).

Sensitivity analyses were performed refitting all models using a less restrictive threshold of 0.1 for retaining autoregressive parameters. Some models changed slightly, retaining additional autoregressive parameters. However, the parameter estimates for the immediate effect of the intervention (*ICD10CM*) and the difference in slopes before and after the intervention *(time-after-ICD10CM*) that were significant in the originally fitted segmented regression models (i.e. had associated *p*-values< 0.05 as reported in Table 1 and Figs. 1 and 2) remained significant in the models ran in the sensitivity analysis. The sensitivity analysis did not result in models with significant parameter estimates for *ICD10CM* or *time-after-ICD10CM* that were not already identified in the original models.

## Discussion

Overall, the proposed ICD-10-CM injury reporting framework provided a reasonably smooth transition from ICD-9-CM. Transitory disruption immediately after the transition was observed in trends of 1) ECOI completeness, 2) percentage of injuries coded as unintentional or undetermined intent, and 3) rates of injury hospitalizations due to poisonings, suffocation, struck by/against, other transportation, other specified NEC, or unspecified mechanism.

Most of these changes were expected. The decline in ECOI completeness was anticipated based on preliminary estimates of decline in coding productivity after the transition to ICD-10-CM coding (Weems et al. 2015). In a survey of Kentucky medical coders, 79% reported that it took longer to code similar cases in ICD-10-CM than in ICD-9-CM (Costich et al. 2017). We found that the drop in ECOI completeness in Kentucky hospital discharge data was significant but lasted only during the first month after ICD-10-CM implementation. When the observation for October 2015 was omitted (Fig. 1b), segmented regression analysis found no significant changes in ECOI completeness level. This is an important result that provides assurance that any notable decreases in injury rates by mechanism or intent of injury after the first transitional month are not due to a decline in data quality.

The significant immediate increase (3.34%) in percentage of injury hospitalizations coded as unintentional injuries in October 2015, and the immediate drop (− 3.39%) in percentage of injury records coded as undetermined intent the same month were expected and aligned with the new ICD-10-CM coding guidelines for injury intent coding. ICD-10-CM Guidelines (PMIC 2016) stated “*If the intent (accident, self-harm, assault) of the cause of an injury or other condition is unknown or unspecified, code the intent as accidental intent. All transport accident categories assume accidental intent. External cause codes for events of undetermined intent are only for use if the documentation in the record specifies that the intent cannot be determined*” (Chapter 20). The steady increase in assault injuries after October 2015, however, was not related to the transition to ICD-10-CM. As we note in the Results section and in Additional file 2, a significant increase in Kentucky assault injury hospitalizations began early in 2015, and this trend is captured by other surveillance data sources. The age-adjusted rate of Kentucky homicides (captured by death certificate data) increased from 4.6/100,000 in 2014 to 6.9/100,000 in 2016 (Bush and Bunn 2018). A query of the CDC WISQARS database (https://www.cdc.gov/injury/wisqars/) found a steady increase in the national age-adjusted rate of homicides over the last 3 years (5.05/100,000 in 2014, 5.64/100,000 in 2015, 6.13/100,000 in 2016) after several years of decline (from 6.20/100,000 in 2006).

The immediate increase in the rate of poisonings and suffocation hospitalizations is likely due to changes in coding structure: in ICD-9-CM, a poisoning or a suffocation was identified by an ECOI code (CDC 2011**)**, whereas in ICD-10-CM, these injuries are identified by diagnosis codes (Annest et al. 2014; CDC/NCHS 2016). Because diagnosis codes are required for reimbursement, while ECOI codes are not, implementation of these ICD-10-CM coding rules was intended to improve identification and capturing of poisoning and suffocation injuries.

The immediate increase in the rates due to “struck by/against” may be attributable to the new category “colliding with stationary object” (V00.112, V00.122, V00.132, etc). There were no codes for “colliding” in ICD-9-CM; these injuries were coded in the range E880- E888, categorized under “falls” in the ICD-9-CM-based external-cause-of-injury matrix. Injuries from “striking against with subsequent fall” (ICD-10-CM codes W18.00×, W18.01×, W18.02×, W18.09) were classified as “falls” in the ICD-9-CM injury reporting framework (E888.1 and E888.0).

According to the ICD-10-CM Guidelines for Coding and Reporting, “codes titled ‘other’ or ‘other specified’ are for use when the information in the medical record provides detail for which a specific code does not exist” (PMIC 2016). The guidelines also state: “Codes titled ‘unspecified’ are for use when the information in the medical record is insufficient to assign a more specific code. For those categories for which an unspecified code is not provided, the ‘other specified’ code may represent both other and unspecified.” The high level of specificity in the ICD-10-CM coding system, as expected, significantly reduced the number of injuries with “other specified, NEC” mechanism, but our results showed a significant initial jump in “unspecified mechanism”. Increased coding specificity requires a higher level of detail in the medical record, a requirement that may not have been well understood by clinical providers in the transition to ICD-10-CM coding.

Medical coders are only allowed to use the clinical provider notes when assigning diagnosis codes (PMIC 2016). When providers do not transfer information from other parts of the medical record (e.g., imaging results, lab results, chief complaint notes) to their notes, medical coders cannot use the information, even if they see it in the medical record. ICD-10-CM coding guidelines (Section I; A. Conventions for the ICD-10-CM; 19. Code assignment and clinical criteria) affirm that” [t]he assignment of a diagnosis code is based on the provider’s diagnostic statement that the condition exists. The provider’s statement that the patient has a particular condition is sufficient. Code assignment is not based on clinical criteria used by the provider to establish the diagnosis “(PMIC 2016). A survey of Kentucky medical coders identified the need for more detailed and specific physician documentation and suggested that physicians (and other clinical providers) should be trained to include more detailed information in their notes (Costich et al. 2017). Future discussions with physician leadership and professional associations should identify efficient ways to improve documentation.

One limitation of our study is that data presented here reflect instances of inpatient hospitalizations, rather than distinct injured patients or injuries. State data policy requires removal of personal identifiers from state administrative billing data sets, so transfers from one hospital to another or readmission for active treatment of the same injury cannot be identified.

Another limitation of our study is that we did not have an appropriate control group. However, as noted by Wagner et al., “even without a control group, segmented regression analysis addresses important threats to internal validity (such as history and maturation) by making multiple assessments of the outcome variable both before and after the intervention” (Wagner et al. 2002).

Further research is needed to determine if the significant changes in the trend of some injury mechanisms (e.g., cut/pierce, firearm, motor vehicle traffic, and other transportation) are a reflection of actual changes in the incidence of these hospitalizations or a result of improved case documentation and coding. Beyond the scope of this study are reasons for observed changes in trends, such as policy changes, seasonality, relevant/competing interventions, etc. The use of autoregressive model with automatic variable selection partially accounted for these injury specific factors. Medical record review studies as well as dual-coding studies are needed to validate the hypotheses generated by the epidemiological trend analyses in this study. Injury surveillance programs should monitor the changes in injury hospitalization trends to determine whether these changes are sustained after the transition period during which medical coders and providers adjusted to the new requirements for documenting and coding injury hospitalization encounters. Gibson and colleagues expressed concern that “after a change in codes, coding rules, or code sets, it may be difficult to separate the impact of transitioning to ICD-10-CM/PCS (Procedure Classification System) from actual changes over time” (Gibson et al. 2016). Epidemiologists may benefit from using external data sources for comparison to determine whether consistent changes in injury trends after October 2015 represent actual changes in injury incidence.

The methodology (segmented regression analysis for interrupted time series) presented in this paper is applicable for evaluation of the effect of the ICD-10-CM transition on any health condition trend (not just injuries), using administrative billing data for inpatient hospitalizations, emergency department visits, or other health care setting administrative claims utilizing ICD-9-CM and ICD-10-CM coded data. The case selection of injury hospitalization encounters of care used in this study are based on consensus definitions for hospital injury surveillance and, therefore, are applicable to other state, facility-specific, or national studies on injury hospitalization trends. However, some results from Kentucky on the transition to ICD-10-CM coding may not be applicable to all states. For example, external-cause-of-injury codes are not mandatory in Kentucky. Therefore, we experienced immediate yet transitory drop in the ECOI completeness during the transition months, which may not be observed in states with mandatory ECOI reporting (e.g., Maryland, Massachusetts). We hope that the SAS code and the sample data set provided in Additional file 1 of this paper will facilitate more studies in different health care settings, populations, and geographical regions, stimulate discussion, and improve our understanding on expected changes versus changes indicating new injury incidence trends.

## Conclusions

The CDC ‘s *Proposed Framework for Presenting Injury Data using ICD-10-CM External Cause of Injury Codes* succeeded in guiding a relatively smooth transition from the ICD-9-CM-based matrix on injury hospitalization trends by intent and mechanism. Our findings are intended to raise awareness among researchers and public health practitioners regarding structural and conceptual changes in the ICD-10-CM coding system that are important for accurate interpretation of changes in injury hospitalization trends by intent and mechanism.

## Additional files

Additional file 1: Sample data set, SAS code, and SAS output for the modeling of the Kentucky resident poisoning hospitalization rates, January 2012 – December 2017. (DOCX 187 kb)

Additional file 2: Kentucky Resident Assault Injury Hospitalizations, January 2012 - December 2017. (PDF 57 kb)

## Abbreviations

CDC: The Centers for Disease Control and Prevention
ECOI: External-cause-of-injury
ICD-10-CM: International Classification of Diseases, Tenth Revision, Clinical Modification
ICD-9-CM: International Classification of Diseases, Ninth Revision, Clinical Modification
MVT: Motor vehicle traffic
