# Supplementary material for: Interrupted time series design to evaluate the effect of the ICD-9-CM to ICD-10-CM coding transition on injury hospitalization trends
Source: Inj Epidemiol. 2018 Oct 1;5:36. doi: 10.1186/s40621-018-0165-8 (PMC6165830; doi:10.1186/s40621-018-0165-8)

**Additional file 1: Sample data set, SAS code, and SAS output for the modeling of the Kentucky resident poisoning hospitalization rates, January 2012 – December 2017.**

**Sample Data Set:**

**Table 1: Data for Segmented Regression Analysis of the Effect of the Transition to ICD-10-CM coding on the Kentucky Resident Rate of Poisoning Hospitalizations**

| Observation | Time (month) | Rate of poisoning hospitalizations per 100,000 residents | Transition to ICD-10-CM | Time after transition to ICD-10-CM |
| --- | --- | --- | --- | --- |
| 2012_01 | 1 | 8.96 | 0 | 0 |
| 2012_02 | 2 | 10.10 | 0 | 0 |
| 2012_03 | 3 | 10.06 | 0 | 0 |
| 2012_04 | 4 | 9.37 | 0 | 0 |
| 2012_05 | 5 | 10.17 | 0 | 0 |
| 2012_06 | 6 | 9.78 | 0 | 0 |
| 2012_07 | 7 | 10.03 | 0 | 0 |
| 2012_08 | 8 | 9.35 | 0 | 0 |
| 2012_09 | 9 | 9.37 | 0 | 0 |
| 2012_10 | 10 | 8.71 | 0 | 0 |
| 2012_11 | 11 | 8.03 | 0 | 0 |
| 2012_12 | 12 | 8.30 | 0 | 0 |
| 2013_01 | 13 | 7.41 | 0 | 0 |
| 2013_02 | 14 | 6.93 | 0 | 0 |
| 2013_03 | 15 | 7.68 | 0 | 0 |
| 2013_04 | 16 | 8.00 | 0 | 0 |
| 2013_05 | 17 | 7.86 | 0 | 0 |
| 2013_06 | 18 | 9.09 | 0 | 0 |
| 2013_07 | 19 | 9.16 | 0 | 0 |
| 2013_08 | 20 | 9.54 | 0 | 0 |
| 2013_09 | 21 | 7.54 | 0 | 0 |
| 2013_10 | 22 | 7.39 | 0 | 0 |
| 2013_11 | 23 | 7.68 | 0 | 0 |
| 2013_12 | 24 | 7.41 | 0 | 0 |
| 2014_01 | 25 | 7.05 | 0 | 0 |
| 2014_02 | 26 | 6.78 | 0 | 0 |
| 2014_03 | 27 | 8.00 | 0 | 0 |
| 2014_04 | 28 | 7.80 | 0 | 0 |
| 2014_05 | 29 | 8.32 | 0 | 0 |
| 2014_06 | 30 | 8.02 | 0 | 0 |
| 2014_07 | 31 | 8.23 | 0 | 0 |
| 2014_08 | 32 | 8.54 | 0 | 0 |
| 2014_09 | 33 | 8.20 | 0 | 0 |
| 2014_10 | 34 | 7.77 | 0 | 0 |
| 2014_11 | 35 | 7.25 | 0 | 0 |
| 2014_12 | 36 | 7.73 | 0 | 0 |
| 2015_01 | 37 | 7.39 | 0 | 0 |
| 2015_02 | 38 | 6.89 | 0 | 0 |
| 2015_03 | 39 | 8.11 | 0 | 0 |
| 2015_04 | 40 | 8.68 | 0 | 0 |
| 2015_05 | 41 | 8.38 | 0 | 0 |
| 2015_06 | 42 | 8.41 | 0 | 0 |
| 2015_07 | 43 | 9.20 | 0 | 0 |
| 2015_08 | 44 | 9.67 | 0 | 0 |
| 2015_09 | 45 | 8.93 | 0 | 0 |
| 2015_10 | 46 | 9.38 | 1 | 1 |
| 2015_11 | 47 | 8.36 | 1 | 2 |
| 2015_12 | 48 | 8.18 | 1 | 3 |
| 2016_01 | 49 | 8.09 | 1 | 4 |
| 2016_02 | 50 | 8.29 | 1 | 5 |
| 2016_03 | 51 | 9.08 | 1 | 6 |
| 2016_04 | 52 | 8.56 | 1 | 7 |
| 2016_05 | 53 | 8.54 | 1 | 8 |
| 2016_06 | 54 | 9.38 | 1 | 9 |
| 2016_07 | 55 | 9.11 | 1 | 10 |
| 2016_08 | 56 | 8.99 | 1 | 11 |
| 2016_09 | 57 | 8.97 | 1 | 12 |
| 2016_10 | 58 | 8.07 | 1 | 13 |
| 2016_11 | 59 | 7.98 | 1 | 14 |
| 2016_12 | 60 | 8.27 | 1 | 15 |
| 2017_01 | 61 | 7.88 | 1 | 16 |
| 2017_02 | 62 | 8.21 | 1 | 17 |
| 2017_03 | 63 | 8.88 | 1 | 18 |
| 2017_04 | 64 | 8.39 | 1 | 19 |
| 2017_05 | 65 | 8.73 | 1 | 20 |
| 2017_06 | 66 | 9.49 | 1 | 21 |
| 2017_07 | 67 | 7.97 | 1 | 22 |
| 2017_08 | 68 | 8.53 | 1 | 23 |
| 2017_09 | 69 | 7.41 | 1 | 24 |
| 2017_10 | 70 | 7.65 | 1 | 25 |
| 2017_11 | 71 | 8.08 | 1 | 26 |
| 2017_12 | 72 | 7.03 | 1 | 27 |

**SAS Code:**

ODS GRAPHICS ON;

**PROC** **AUTOREG** DATA = injurytrends;

MODEL poison_rate = time icd10cm time_after_icd10cm

/PLOTS=ALL METHOD=ML NLAG=**12** DWPROB BACKSTEP;

OUTPUT OUT=outputdata PM=trendhat LCLM=lclm UCLM=uclm P=yhat LCL=lcl UCL=ucl;

/*PM= predicted values from only the structural part of the model;

useful in predicting the trend */;

/*P= predicted values from both the structural and autoregressive parts of the model;

useful in predicting future values of the current response time series */;

**RUN**;

**PROC** **SGPLOT** DATA=outputdata;

SCATTER X=time Y=poison_rate / MARKERATTRS = (COLOR=BLACK)

LEGENDLABEL = "Observed values";

SERIES X=time Y=yhat / LINEATTRS =(COLOR=DABGR PATTERN=SHORTDASH)

LEGENDLABEL = "Predicted series values";

BAND X=time UPPER=ucl LOWER=lcl / FILLATTRS=(COLOR=LTGREY TRANSPARENCY=**.7**)

LEGENDLABEL = "Band for predicted series values";

SERIES X=time Y=trendhat / LINEATTRS =(COLOR= BLACK PATTERN=SOLID)

LEGENDLABEL = "Predicted mean (trend)";

BAND X=time UPPER=uclm LOWER=lclm /

FILLATTRS= (COLOR= MEGR TRANSPARENCY=**.5**)

LEGENDLABEL = "Band for predicted trend";

LABEL poison_rate = "Rate of poisoning hospitalizations per 100,000 residents"

time ="Month";

REFLINE **46** / AXIS = X LINEATTRS =(PATTERN=SHORTDASH COLOR=BLACK);

**RUN**;

ODS GRAPHICS OFF;

**SAS Output:**

**Figure 1. Segmented Regression Analysis of Kentucky Resident Poisoning Hospitalization Rate, 2012 – 2017**

| ***The AUTOREG Procedure*** | |  |  |
| --- | --- | --- | --- |
| **Dependent Variable** | | poison_rate | |
|  | | poison_rate | |

| **Ordinary Least Squares Estimates** | | | |
| --- | --- | --- | --- |
| **SSE** | 41.6593671 | **DFE** | 68 |
| **MSE** | 0.61264 | **Root MSE** | 0.78271 |
| **SBC** | 182.039742 | **AIC** | 172.933078 |
| **MAE** | 0.63348842 | **AICC** | 173.530093 |
| **MAPE** | 7.62824877 | **HQC** | 176.558468 |
| **Durbin-Watson** | 0.7010 | **Regress R-Square** | 0.1567 |
|  |  | **Total R-Square** | 0.1567 |

| **Durbin-Watson Statistics** | | | |
| --- | --- | --- | --- |
| **Order** | **DW** | **Pr < DW** | **Pr > DW** |
| **1** | 0.7010 | <.0001 | 1.0000 |

| ***NOTE: Pr<DW is the p-value for testing positive autocorrelation, and Pr>DW is the p-value for testing negative autocorrelation.*** |
| --- |

| **Parameter Estimates** | | | | | | |
| --- | --- | --- | --- | --- | --- | --- |
| **Variable** | **DF** | **Estimate** | **Standard Error** | **t Value** | **Approx Pr > \|t\|** | **Variable Label** |
| **Intercept** | 1 | 9.0200 | 0.2373 | 38.01 | <.0001 |  |
| **time** | 1 | -0.0277 | 0.008984 | -3.08 | 0.0030 | time |
| **icd10cm** | 1 | 1.1277 | 0.3856 | 2.92 | 0.0047 | icd10cm |
| **time_after_icd10cm** | 1 | -0.006403 | 0.0213 | -0.30 | 0.7649 | time_after_icd10cm |

| **Estimates of Autocorrelations** | | | |
| --- | --- | --- | --- |
| **Lag** | **Covariance** | **Correlation** | -1 9 8 7 6 5 4 3 2 1 0 1 2 3 4 5 6 7 8 9 1 |
| **0** | 0.5786 | 1.000000 | \| \|********************\| |
| **1** | 0.3695 | 0.638580 | \| \|************* \| |
| **2** | 0.2416 | 0.417605 | \| \|******** \| |
| **3** | 0.1332 | 0.230234 | \| \|***** \| |
| **4** | -0.0210 | -0.036297 | \| *\| \| |
| **5** | -0.1137 | -0.196484 | \| ****\| \| |
| **6** | -0.2046 | -0.353644 | \| *******\| \| |
| **7** | -0.1646 | -0.284530 | \| ******\| \| |
| **8** | -0.0933 | -0.161315 | \| ***\| \| |
| **9** | -0.00305 | -0.005271 | \| \| \| |
| **10** | 0.0770 | 0.133081 | \| \|*** \| |
| **11** | 0.1643 | 0.284022 | \| \|****** \| |
| **12** | 0.1763 | 0.304673 | \| \|****** \| |

| **Backward Elimination of Autoregressive Terms** | | | |
| --- | --- | --- | --- |
| **Lag** | **Estimate** | **t Value** | **Pr > \|t\|** |
| **8** | 0.026135 | 0.17 | 0.8659 |
| **2** | -0.041636 | -0.29 | 0.7716 |
| **5** | -0.065031 | -0.45 | 0.6529 |
| **10** | 0.069503 | 0.48 | 0.6351 |
| **7** | -0.107862 | -0.82 | 0.4141 |
| **12** | 0.114409 | 0.91 | 0.3677 |
| **11** | -0.094324 | -0.89 | 0.3793 |
| **4** | 0.157947 | 1.28 | 0.2066 |
| **3** | -0.155565 | -1.35 | 0.1809 |
| **9** | -0.155269 | -1.68 | 0.0970 |

| **Preliminary MSE** | 0.3113 |
| --- | --- |

| **Estimates of Autoregressive Parameters** | | | |
| --- | --- | --- | --- |
| **Lag** | **Coefficient** | **Standard Error** | **t Value** |
| **1** | -0.591947 | 0.092086 | -6.43 |
| **6** | 0.237335 | 0.092086 | 2.58 |

| **Expected Autocorrelations** | |
| --- | --- |
| **Lag** | **Autocorr** |
| **0** | 1.0000 |
| **1** | 0.6248 |
| **2** | 0.3659 |
| **3** | 0.1750 |
| **4** | 0.0168 |
| **5** | -0.1384 |
| **6** | -0.3192 |

| Algorithm converged. |
| --- |

| **Maximum Likelihood Estimates** | | | |
| --- | --- | --- | --- |
| **SSE** | 21.493076 | **DFE** | 66 |
| **MSE** | 0.32565 | **Root MSE** | 0.57066 |
| **SBC** | 144.158017 | **AIC** | 130.49802 |
| **MAE** | 0.44306871 | **AICC** | 131.790328 |
| **MAPE** | 5.32765357 | **HQC** | 135.936105 |
| **Log Likelihood** | -59.24901 | **Regress R-Square** | 0.1360 |
| **Durbin-Watson** | 1.9218 | **Total R-Square** | 0.5649 |
|  |  | **Observations** | 72 |

| **Durbin-Watson Statistics** | | | |
| --- | --- | --- | --- |
| **Order** | **DW** | **Pr < DW** | **Pr > DW** |
| **1** | 1.9218 | 0.2524 | 0.7476 |

| ***NOTE: Pr<DW is the p-value for testing positive autocorrelation, and Pr>DW is the p-value for testing negative autocorrelation.*** |
| --- |

| **Parameter Estimates** | | | | | | |
| --- | --- | --- | --- | --- | --- | --- |
| **Variable** | **DF** | **Estimate** | **Standard Error** | **t Value** | **Approx Pr > \|t\|** | **Variable Label** |
| **Intercept** | 1 | 8.8873 | 0.2699 | 32.93 | <.0001 |  |
| **time** | 1 | -0.0252 | 0.0103 | -2.45 | 0.0171 | time |
| **icd10cm** | 1 | 1.2895 | 0.4451 | 2.90 | 0.0051 | icd10cm |
| **time_after_icd10cm** | 1 | -0.0160 | 0.0248 | -0.64 | 0.5213 | time_after_icd10cm |
| **AR1** | 1 | -0.5774 | 0.0937 | -6.16 | <.0001 |  |
| **AR6** | 1 | 0.2791 | 0.0955 | 2.92 | 0.0047 |  |

| **Expected Autocorrelations** | |
| --- | --- |
| **Lag** | **Autocorr** |
| **0** | 1.0000 |
| **1** | 0.6274 |
| **2** | 0.3641 |
| **3** | 0.1644 |
| **4** | -0.0067 |
| **5** | -0.1790 |
| **6** | -0.3824 |

| **Autoregressive parameters assumed given** | | | | | | |
| --- | --- | --- | --- | --- | --- | --- |
| **Variable** | **DF** | **Estimate** | **Standard Error** | **t Value** | **Approx Pr > \|t\|** | **Variable Label** |
| **Intercept** | 1 | 8.8873 | 0.2665 | 33.35 | <.0001 |  |
| **time** | 1 | -0.0252 | 0.0100 | -2.51 | 0.0146 | time |
| **icd10cm** | 1 | 1.2895 | 0.4299 | 3.00 | 0.0038 | icd10cm |
| **time_after_icd10cm** | 1 | -0.0160 | 0.0248 | -0.65 | 0.5211 | time_after_icd10cm |


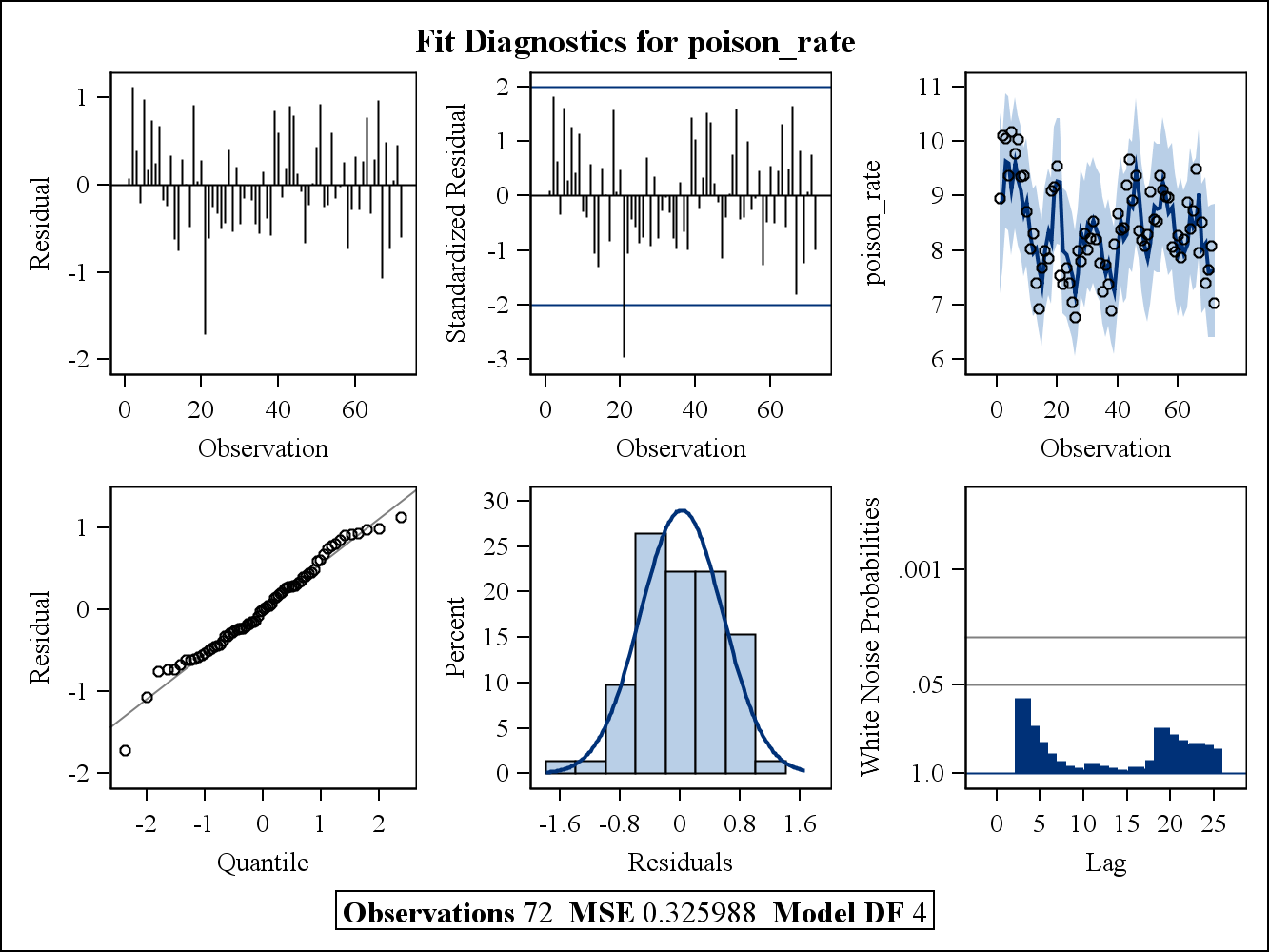


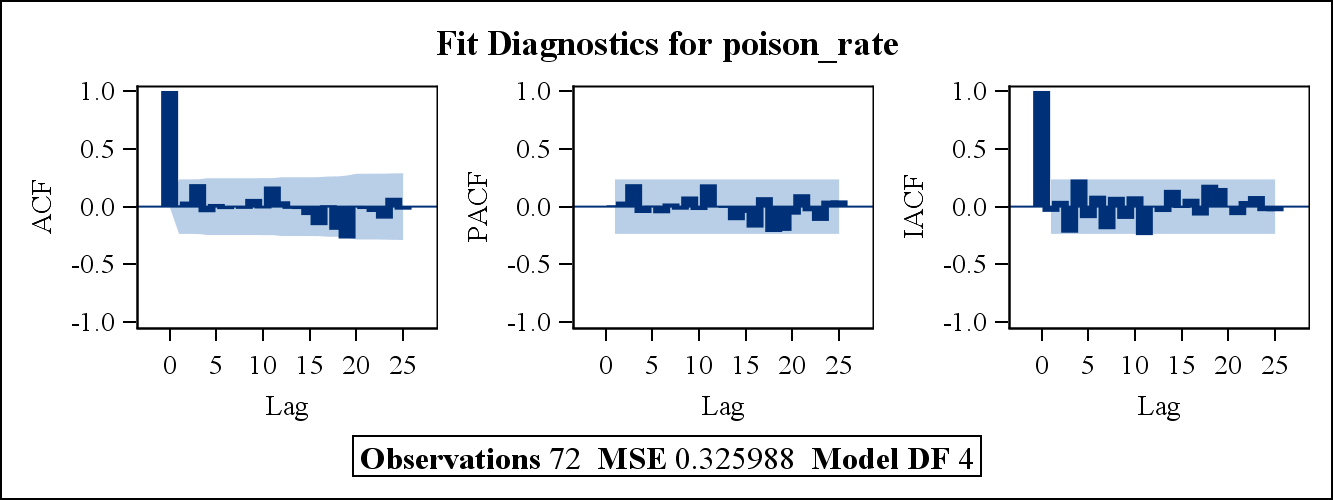

Supplement: Supplementary file 1 — Sample data set, SAS code, and SAS output for the modeling of the Kentucky resident poisoning hospitalization rates, January 2012 – December 2017. (DOCX 187 kb) [file 40621_2018_165_MOESM1_ESM.docx]
