# Supplementary material for: Interrupted time series design to evaluate the effect of the ICD-9-CM to ICD-10-CM coding transition on injury hospitalization trends
Source: Inj Epidemiol. 2018 Oct 1;5:36. doi: 10.1186/s40621-018-0165-8 (PMC6165830; doi:10.1186/s40621-018-0165-8)
Supplement: Supplementary file 2 — Kentucky Resident Assault Injury Hospitalizations, January 2012 - December 2017. (PDF 57 kb) [file 40621_2018_165_MOESM2_ESM.pdf]

Additional file 2: Kentucky Resident Assault Injury Hospitalizations, January 2012 - December 2017

A) Number of Assault Injury Hospitalizations, Kentucky 2012 – 2017 Join Point in October 2015

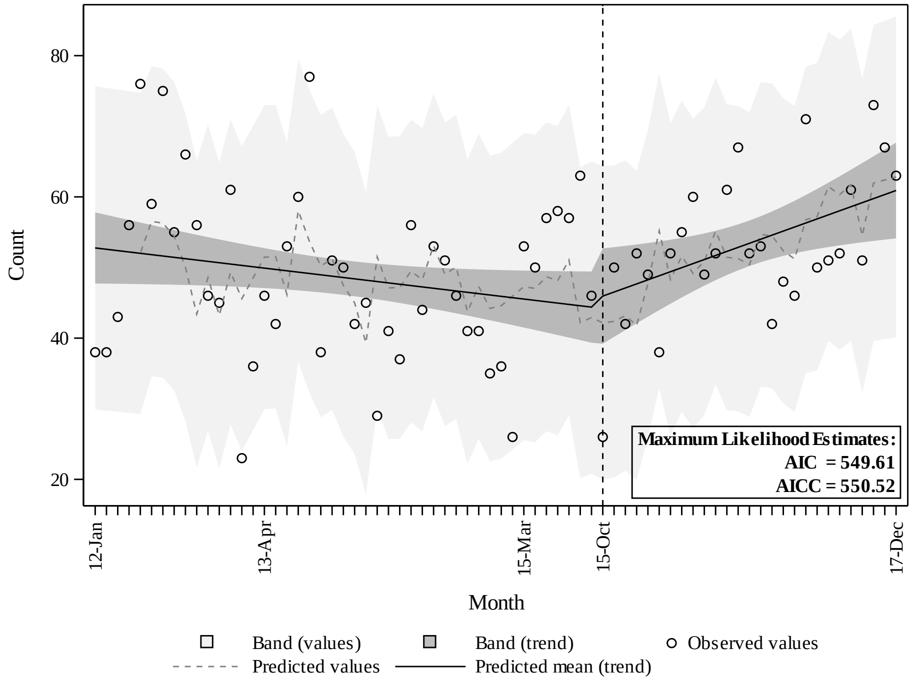

Variable	*Parameter Estimate**	*P value*
Intercept	52.96	<.0001
Time (month)	-0.19	.0601
Transition to ICD-10-CM effect (Oct 2015)	0.98	.8281
Time after Oct 2015	0.77	.0022

B) Number of Assault Injury Hospitalizations, Kentucky 2012 – 2017 Join Point in March 2015

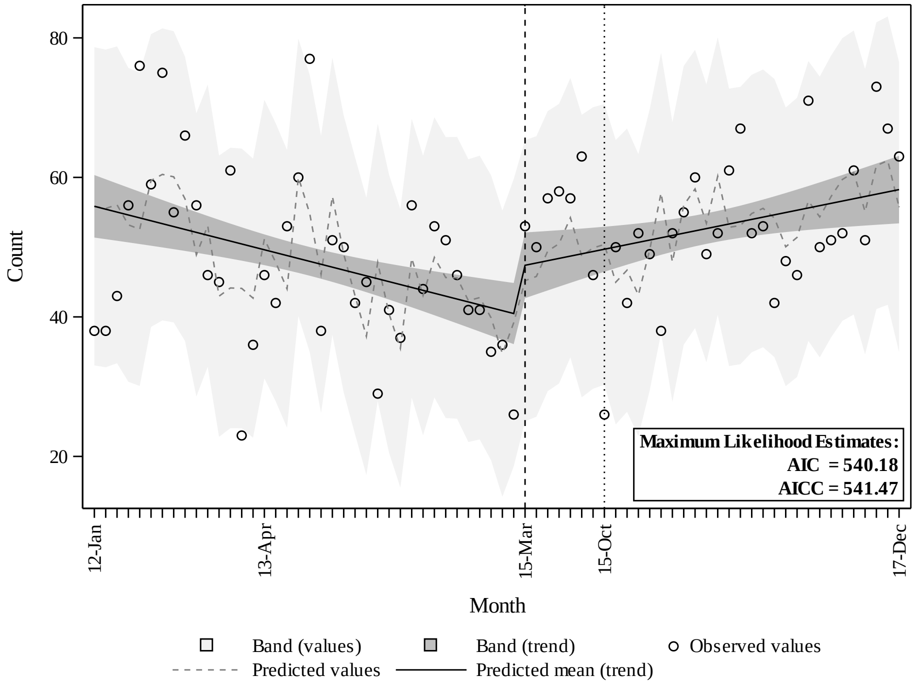

Variable	*Parameter Estimate**	*P value*
Intercept	56.27	<.0001
Time (month)	-0.42	.0002
Join point effect (Mar 2015)	6.59	.0700
Time after Mar 2015	0.74	<.0001

*Autoregressive parameter(s) assumed given
